# Adverse event reporting of four anti-Calcitonin gene-related peptide monoclonal antibodies for migraine prevention: a real-world study based on the FDA adverse event reporting system

**DOI:** 10.3389/fphar.2023.1257282

**Published:** 2024-01-09

**Authors:** Wenfang Sun, Yali Li, Binbin Xia, Jing Chen, Yang Liu, Jingyao Pang, Fang Liu, Hua Cheng

**Affiliations:** Department of Pharmacy, Beijing Luhe Hospital, Capital Medical University, Beijing, China

**Keywords:** Calcitonin gene-related peptide, adverse events, migraine, FDA adverse events reporting system, safety

## Abstract

**Background:** Anti-Calcitonin gene-related peptide monoclonal antibodies (anti-CGRP mAbs) have shown significant efficacy in preventing migraine. However, there have been limited reports of adverse events (AEs) after marketing, particularly for eptinezumab launched in 2020. The study aimed to mine and analyze the AE signals with four anti-CGRP mAbs from the United States Food and Drug Administration (FDA) Adverse Event Reporting System (FAERS) database to gain insights into the safety profile of these medications post-marketing.

**Methods:** All AE reports on the four anti-CGRP mAbs (erenumab, galcanezumab, fremanezumab, and eptinezumab) were retrieved from the FAERS database from the first quarter (Q1) of 2018 to Q1 of 2023. Disproportionality analysis was measured by reporting odd ratio (ROR) and Bayesian confidence propagation neural network (BCPNN) to identify potential AE signals. Comparisons were made between the four drugs in terms of AEs.

**Results:** A total of 38,515 reports of erenumab, 19,485 reports of galcanezumab, 5,332 reports of fremanezumab, and 2,460 reports of eptinezumab were obtained, mostly reported in the second to third year after launch in the market. The common AEs to erenumab included constipation (17.93%), injection site pain (14.08%), and alopecia (7.23%). The AEs that occurred more frequently with galcanezumab included injection site pain (24.37%), injection site erythema (5.35%), and injection site haemorrhage (4.97%). Common AEs related to fremanezumab were injection site pain (13.10%), injection site erythema (7.02%), and injection site pruritus (5.47%). Fatigue (13.54%), throat irritation (9.02%), and pruritus (8.20%) were the most common AEs with eptinezumab. In addition, there are new AEs that were not listed in the drug instructions but occurred concurrently with multiple drugs, such as Raynaud’s phenomenon, weight increase, menstrual disorders, throat tightness, and paraesthesia oral.

**Conclusion:** Common AE signals of the four anti-CGRP mAbs and new AE signals were found to provide a reference for clinical drug selection in clinical practice.

## 1 Introduction

Calcitonin gene-related peptide (CGRP), a peptide neurotransmitter, and its receptors are widely distributed in the trigeminal vascular system and the central nervous system (Liu et al., 2022). The release of CGRP increases during migraine attacks, and CGRP levels are positively correlated with headache severity (Goadsby et al., 1988). Four monoclonal antibodies (mAbs) targeting the CGRP have been approved by the United States Food and Drug Administration (FDA) for the prevention of episodic and chronic migraine, including one anti-CGRP receptor mAb (Eerenumab) and 2 anti-CGRP ligand mAbs (fremanezumab and galcanezumab) available in 2018 and 1 anti-CGRP ligand mAb (eptinezumab) available in 2020. These mAbs can significantly prevent episodic or chronic migraine, as shown by reduced numbers of migraine days per month and days on acute medication, with a good safety profile.

Currently, due to the better preventive effect of CGRP antibodies and the cyclical nature of migraine attacks, German and European guidelines recommend that migraine patients undergo a treatment break after 9–12 months of CGRP antibody therapy (Diener et al., 2020). However, current real-world data suggests that migraine headaches will appear an increasing deteriorating trend during the 3 months of discontinuing CGRP antibodies in most patients (Pavelic et al., 2022). More data are needed on the benefits of treatment interruption.

The majority of studies support good effectiveness and tolerability of anti-CGRP-mAbs in the real world (Pavelic et al., 2022). However, there is not much data on these drugs’ post-marketing safety, and many available papers are real-world single-center studies with limited sample sizes (Alex et al., 2020; Kanaan et al., 2020; Viudez-Martinez et al., 2022). Furthermore, since eptinezumab is a newly marketed anti-CGRP mAb, there are few reports of relevant adverse events (AEs). By comparing the AEs of other anti-CGRP mAbs, the potential AEs of eptinezumab might be identified more quickly and provide recommendations for clinical use.

The FDA Adverse Event Reporting System (FAERS) is an important source of data about AEs in the real-world setting. The FAERS database is a public, voluntary, and spontaneous reporting system that contains information on AEs and medication error reports submitted by health professionals, consumers, and drug manufacturers, thus reflecting, to some extent, the occurrence of drug AEs in the real world.

Therefore, this study aimed to mine AEs on the four anti-CGRP mAbs for migraine prophylaxis from the FAERS database. By comparing the similarities and differences of AEs among four anti-CGRP mAbs, undetected AEs were explored to provide forewarning for clinical drug selection. The results should provide reference to clinicians and promote further research in the real world.

## 2 Methods

### 2.1 Data source

The FAERS database was summarized quarterly and contains AE reports, medication errors, and product quality issues. As erenumab, fremanezumab and galcanezumab were all launched in 2018, the data retrieval started from the first quarter (Q1) of 2018 to Q1 of 2023, and a total of 21 quarterly ASCII data packages were extracted from the FAERS database and imported into the SAS 9.4 software for data cleaning and analysis. Data were cleaned by deduplication and excluding missing values. According to the FDA’s recommendations, we selected the latest FDA_DT (date FDA received the case) when the PRIMARYIDs (a unique number for identifying a FAERS report) were the same, and chose the highest PRIMARYID when the FDA_DT and the CASEID (a number for identifying a FAERS case) were the same, to remove duplicate reports submitted by various individuals and institutions. FAERS reported drugs are arbitrary, so the generic names and brand names were used as keywords for data extraction. The AEs were classified and standardized based on the preferred terms (PTs) and system organ classes (SOCs) in the Medical Dictionary for Regulatory Activities (MedDRA).

### 2.2 Data mining and analysis

Disproportionality analysis was performed in our study to indicate the proportion of AEs occurring between a specific drug and all other drugs. Two disproportional signal detection methods used in this study were reporting odd ratio (ROR) and Bayesian confidence propagation neural network (BCPNN). These methods were based on the two-by-two contingency table, if the ratio exceeds the specified threshold, i.e., the ratio is out of proportion, it indicates signal generation (Huang et al., 2014). The corresponding ROR, information components (IC), and 95% confidence interval (CI) were calculated accordingly to determine the signal intensity of each adverse event for each drug. The calculation formulas are shown in Table 1.

**TABLE 1:** Calculation formulas.

	AEs of interest	All other AEs	Total
Drugs of interest	a	b	a+b
All other drugs in FAERs	c	d	c + d
Total	a+c	b + d	N = a+b + c + d

Note: FAERS: FDA adverse event reporting system; AEs: adverse events.

Reporting odds ratio (ROR) = $\frac{\text{ad}}{\text{bc}}$ .

ROR $95\%\text{CI}=e^{\ln\left(\text{ROR}\right)\pm 1.96\sqrt{\;\left(\frac{1}{a}+\frac{1}{b}+\frac{1}{c}+\frac{1}{d}\right)\;}}$

Information components (IC) = $\log_{2}\frac{a\left(a+b+c+d\right)}{\left(a+b\right)\left(a+c\right)}$

IC_025_ = $e^{\ln\left(\text{IC}\right)‐1.96\;\left(\frac{1}{a}+\frac{1}{b}+\frac{1}{c}+\frac{1}{d}\right)\;*0.5}$

To generate a valid signal in screening, the number of reports should be at least 3, the lower limit of ROR 95% CI should be greater than one, and IC_025_ must be above 0. An association between the AE and the target drug was demonstrated by valid signal generation. A larger signal value (i.e., ROR) indicated a stronger association between the target drug and the suspected AE. However, it does not necessarily mean that there was a causal relationship between the two biologically according to FDA instruction, and reports do not have enough detail to evaluate an event properly. In our study, we excluded AEs associated with product problems, medication errors, off-label or unlicensed use, indication-related, and disease states.

## 3 Results

### 3.1 Characteristic of the patients

A total of 65,792 reports for CGRP mAbs have been entered into the FAERS from the Q1 of 2018 to the Q1 of 2023, including 38,515 for erenumab, 19,485 for galcanezumab, 5,332 for fremanezumab, and 2,460 for eptinezumab. Most patients were between 45 and 65 years old, and the average age was 48.66 (14.96). There were more women than men in these reports and the percentages of females in the reports for erenumab, galcanezumab, fremanezumab, and eptinezumab were 68.45%, 76.26%, 81.81%, and 74.96%, respectively. The highest rates of AE reporting were concentrated in the second to third years after the launch of the drugs. The country with the most reported data was the United States (95.33%). The demographic information of the patients treated with the four anti-CGRP mAbs is shown in Table 2.

**TABLE 2:** Demographic information on patients treated with anti-CGRP mAbs.

	Total		Erenumab		Galcanezumab		Fremanezumab		Eptinezumab	
	(n = 65,792)		(n = 38,515)		(n = 19,485)		(n = 5,332)		(n = 2,460)	
	n	%	n	%	n	%	n	%	n	%
Sex										
Male	7,646	11.62	4,736	12.30	2,124	10.90	493	9.25	293	11.91
Female	47,431	72.09	26,365	68.45	14,860	76.26	4,362	81.81	1,844	74.96
Unknown	10,715	16.29	7,414	19.25	2,501	12.84	477	8.95	323	13.13
Age (years)										
<18	278	0.38	202	0.52	43	0.22	29	0.54	4	0.16
18–45	12,163	11.91	7,909	20.53	2,597	13.33	1,000	18.75	657	26.71
45–65	15,456	24.58	10,689	27.75	2,643	13.56	1,112	20.86	1,012	41.14
>65	5,343	6.52	4,067	10.56	632	2.25	383	7.18	261	9.44
Unknown	32,552	56.61	15,648	40.63	13,570	69.64	2,808	52.66	526	21.38
Mean (SD)	48.66 (14.96)		49.84 (15.18)		46.32 (14.42)		48.19 (15.04)		49.59 (13.83)	
Reporting year										
2018	7,427	11.29	7,088	18.40	218	1.12	121	2.27	0	0
2019	16,444	24.99	10,716	27.82	4,499	23.09	1,229	23.05	0	0
2020	15,783	23.99	8,439	21.91	6,351	32.59	869	16.30	124	5.04
2021	12,131	18.44	5,629	14.62	4,408	22.62	1,359	25.49	735	29.88
2022	11,240	17.08	5,335	13.85	3,305	16.96	1,387	26.01	1,213	49.31
2023	2,767	4.21	1,308	3.40	704	3.61	367	6.88	388	15.77
Serious outcomes										
Hospitalization	1,264	2.86	1,271	3.30	456	2.34	289	5.42	67	2.72
Disability	348	0.79	515	1.34	160	0.82	98	1.84	7	0.28
Life-threatening	175	0.40	209	0.54	42	0.22	32	0.60	6	0.24
Death	252	0.57	296	0.77	40	0.21	35	0.66	9	0.37
Reported from the United States	62,721	95.33	36,436	94.60	19,151	98.29	4,703	88.20	2,431	98.82

### 3.2 Signal detection at the SOC for four anti-CGRP mAbs

Based on the disproportionality analysis, the final positive signals for the 4 CGRP antibodies, erenumab, galcanezumab, fremanezumab, and eptinezumab, used for analysis were 105, 103, 97, and 44, respectively, and the numbers of reports were 16,099, 16,736, 5,886, and 1,086, respectively (Table 3). For erenumab, the top three SOCs are general disorders and administration site conditions (n = 6,918, 42.97%), gastrointestinal disorders (n = 3,434, 21.33%), and skin and subcutaneous tissue disorders (n = 1,459, 9.06%). For galcanezumab, AEs are mainly focused on the three SOCs of general disorders and administration site conditions (n = 10,539, 62.97%), skin and subcutaneous tissue disorders (n = 1,701, 10.16%), and psychiatric disorders (n = 1,160, 6.93%). General disorders and administration site conditions (n = 3,456, 58.87%), skin and subcutaneous tissue disorders (n = 885, 15.04%), and psychiatric disorders (n = 336, 5.71%) are the top three SOCs for fremanezumab. Respiratory, thoracic, and mediastinal disorders (n = 323, 29.74%), general disorders and administration site conditions (n = 305, 28.08%), and infections and infestations (n = 92, 8.47%) are the common SOC for eptinezumab. AEs signal detection under each SOC for four anti-CGRP mAbs were shown in Supplementary Table S1–S4.

**TABLE 3:** Signal detection of four anti-CGRP mAbs at the SOC level.

Erenumab				Galcanezumab				Fremanezumab				Eptinezumab			
SOC	PT	n	%	SOC	PT	n	%	SOC	PT	n	%	SOC	PT	n	%
General disorders and administration site conditions	30	6,918	42.97	General disorders and administration site conditions	35	10,539	62.97	General disorders and administration site conditions	35	3,465	58.87	Respiratory, thoracic and mediastinal disorders	11	323	29.74
Gastrointestinal disorders	17	3,434	21.33	Skin and subcutaneous tissue disorders	6	1,701	10.16	Skin and subcutaneous tissue disorders	12	885	15.04	General disorders and administration site conditions	11	305	28.08
Skin and subcutaneous tissue disorders	5	1,459	9.06	Psychiatric disorders	13	1,160	6.93	Psychiatric disorders	11	336	5.71	Infections and infestations	2	92	8.47
Psychiatric disorders	8	1,444	8.97	Gastrointestinal disorders	6	683	4.08	Gastrointestinal disorders	6	247	4.20	Skin and subcutaneous tissue disorders	1	89	8.20
Musculoskeletal and connective tissue disorders	7	1,091	6.78	Nervous system disorders	10	597	3.57	Musculoskeletal and connective tissue disorders	3	223	3.79	Immune system disorders	3	82	7.55
Nervous system disorders	10	616	3.83	Musculoskeletal and connective tissue disorders	6	567	3.39	Nervous system disorders	8	203	3.45	Gastrointestinal disorders	4	59	5.43
Investigations	3	425	2.64	Investigations	3	547	3.27	Investigations	2	144	2.45	Vascular disorders	2	34	3.13
Cardiac disorders	3	267	1.66	Immune system disorders	3	329	1.97	Immune system disorders	1	112	1.90	Nervous system disorders	2	29	2.67
Reproductive system and breast disorders	8	182	1.13	Eye disorders	1	194	1.16	Cardiac disorders	1	85	1.44	Injury, poisoning and procedural complications	3	29	2.67
Injury, poisoning and procedural complications	6	91	0.57	Reproductive system and breast disorders	8	131	0.78	Injury, poisoning and procedural complications	5	63	1.07	Investigations	1	17	1.57
Vascular disorders	2	54	0.34	Cardiac disorders	2	124	0.74	Respiratory, thoracic and mediastinal disorders	4	53	0.90	Eye disorders	2	12	1.10
Respiratory, thoracic and mediastinal disorders	1	46	0.29	Respiratory, thoracic and mediastinal disorders	2	54	0.32	Reproductive system and breast disorders	4	32	0.54	Musculoskeletal and connective tissue disorders	1	9	0.83
Eye disorders	2	34	0.21	Injury, poisoning and procedural complications	3	42	0.25	Vascular disorders	1	14	0.24	Metabolism and nutrition disorders	1	6	0.55
Immune system disorders	1	21	0.13	Vascular disorders	1	35	0.21	Infections and infestations	2	12	0.20				
Endocrine disorders	1	11	0.07	Infections and infestations	3	19	0.11	Eye disorders	2	12	0.20				
Renal and urinary disorders	1	6	0.04	Ear and labyrinth disorders	1	14	0.08								
Total	105	16,099	100.00		103	16,736	100.00		97	5,886	100.00		44	1,086	100.00

Notes:SOC: system organ class; PT: preferred term.

### 3.3 The common AEs for four anti-CGRP mAbs

AEs were ranked according to frequency of occurrence, and the top 30 AEs were listed for each drug in Table 4. The five most common AEs to erenumab included constipation (n = 2,287, 17.93%), injection site pain (n = 2,267,14.08%), alopecia (n = 1,164,7.23%), injection site haemorrhage (n = 926, 5.75%), and muscle spasms (n = 625,3.88%). The AEs that occurred more frequently with galcanezumab included injection site pain (n = 4,079, 24.37%), injection site erythema (n = 896, 5.35%), injection site haemorrhage (n = 831, 4.97%), injection site pruritus (n = 676, 4.04%), injection site swelling (n = 663, 3.96%). Common AEs related to fremanezumab were injection site pain (n = 771, 13.10%), injection site erythema (n = 413, 7.02%), injection site pruritus (n = 322, 5.47%), injection site swelling (n = 262, 4.45%), and pruritus (n = 216, 3.67%). There were fewer signals mined for eptinezumab since it launched later than the three other anti-CGRP mAbs. AEs with an incidence of more than 5% were fatigue (n = 147, 13.54%), throat irritation (n = 98, 9.02%), pruritus (n = 89, 8.20%), nasal congestion (n = 81, 7.46%), feeling abnormal (n = 61, 5.62%), COVID-19 (n = 61, 5.62%), and hypersensitivity (n = 56, 5.16%).

**TABLE 4:** Top 30 AEs for four anti-CGRP mAbs.

	Erenmab			Galcanezumab			Fremanezumab			Eptinezumab		
	AE	n	%	AE	n	%	AE	n	%	AE	n	%
1	Constipation	2,887	17.93	Injection site pain	4,079	24.37	Injection site pain	771	13.10	Fatigue	147	13.54
2	Injection site pain	2,267	14.08	Injection site erythema	896	5.35	Injection site erythema	413	7.02	Throat irritation	98	9.02
3	Alopecia	1,164	7.23	Injection site haemorrhage	831	4.97	Injection site pruritus	322	5.47	Pruritus	89	8.20
4	Injection site haemorrhage	926	5.75	Injection site pruritus	676	4.04	Injection site swelling	262	4.45	Nasal congestion	81	7.46
5	Muscle spasms	625	3.88	Injection site swelling	663	3.96	Pruritus	216	3.67	Feeling abnormal	61	5.62
6	Feeling abnormal	542	3.37	Injection site reaction	595	3.56	Injection site reaction	193	3.28	COVID-19	61	5.62
7	Injection site bruising	535	3.32	Alopecia	582	3.48	Rash	190	3.23	Hypersensitivity	56	5.16
8	Anxiety	444	2.76	Weight increased	528	3.15	Alopecia	182	3.09	Oropharyngeal pain	45	4.14
9	Injection site erythema	437	2.71	Constipation	495	2.96	Injection site rash	170	2.89	Rhinorrhoea	40	3.68
10	Injection site swelling	418	2.60	Injection site bruising	434	2.59	Injection site extravasation	167	2.84	Nasopharyngitis	31	2.85
11	Weight increased	407	2.53	Pruritus	389	2.32	Constipation	159	2.70	Constipation	31	2.85
12	Insomnia	386	2.40	Rash	381	2.28	Arthralgia	146	2.48	Memory impairment	25	2.30
13	Depression	338	2.10	Injection site urticaria	378	2.26	Weight increased	139	2.36	Chest discomfort	24	2.21
14	Myalgia	317	1.97	Anxiety	378	2.26	Urticaria	132	2.24	Infusion site pain	23	2.12
15	Influenza like illness	274	1.70	Feeling abnormal	355	2.12	Injection site mass	131	2.23	Flushing	22	2.03
16	Injection site reaction	257	1.60	Injection site mass	346	2.07	Feeling abnormal	128	2.17	Anaphylactic reaction	21	1.93
17	Urticaria	253	1.57	Arthralgia	335	2.00	Anxiety	122	2.07	Sneezing	20	1.84
18	Hypoaesthesia	253	1.57	Urticaria	296	1.77	Hypersensitivity	112	1.90	Infusion related reaction	19	1.75
19	Paraesthesia	252	1.57	Injection site rash	270	1.61	Injection site urticaria	109	1.85	Heart rate increased	17	1.57
20	Palpitations	245	1.52	Hypersensitivity	261	1.56	Injection site bruising	94	1.60	Throat tightness	13	1.20
21	Injection site pruritus	215	1.34	Insomnia	203	1.21	Insomnia	93	1.58	Dry mouth	13	1.20
22	Abdominal distension	181	1.12	Visual impairment	194	1.16	Injection site haemorrhage	91	1.55	Hot flush	12	1.10
23	Injection site urticaria	158	0.98	Depression	187	1.12	Erythema	85	1.44	Infusion site bruising	11	1.01
24	Injection site rash	134	0.83	Injection site warmth	177	1.06	Palpitations	85	1.44	Infusion site extravasation	9	0.83
25	Injection site mass	120	0.75	Myalgia	148	0.88	Injection site warmth	76	1.29	Paraesthesia oral	9	0.83
26	Injection site extravasation	109	0.68	Paraesthesia	135	0.81	Myalgia	66	1.12	Fibromyalgia	9	0.83
27	Injection site indentation	107	0.66	Hypoaesthesia	125	0.75	Paraesthesia	58	0.99	Pharyngeal swelling	8	0.74
28	Panic attack	85	0.53	Memory impairment	125	0.75	Hypoaesthesia	56	0.95	Sinus congestion	8	0.74
29	Injection site discomfort	70	0.43	Stress	120	0.72	Chest pain	53	0.90	Eye pruritus	8	0.74
30	Irritable bowel syndrome	70	0.43	Palpitations	120	0.72	Injection site discharge	52	0.88	Feeling cold	7	0.64

Notes: AE, adverse event.

### 3.4 AEs co-reported for the four anti-CGRP mAbs

We conducted a comparison of the AEs with the four drugs (Table 5). In addition to injection-related adverse events, 31 AEs were reported in more than three anti-CGRP mAbs. Four AEs have been reported to all four anti-CGRP mAbs, including constipation, feeling abnormal, throat tightness, and paraesthesia oral. There are 21 AEs co-reported in all three subcutaneously administered drugs, including alopecia, anxiety, weight increase, insomnia, myalgia, influenza-like illness, hypoaesthesia, urticaria, paraesthesia, palpitations, abdominal distension, panic attack, fear of injection, menstruation irregular, abnormal dreams, Raynaud’s phenomenon, muscle tightness, menstrual disorder, trichorrhexis, hormone level abnormal, and oligomenorrhoea. Some of the AEs have been reported with eptinezumab, which are also reported in galcanezumab, fremanezumab, and erenumab, including concussion, fibromyalgia, blepharospasm, pruritus, pharyngeal swelling, and swollen tongue.

**TABLE 5:** AEs co-reported for the four anti-CGRP mAbs.

		Erenumab			Galcanezumab			Fremanezumab			Eptinezumab		
	AE	n	%	ROR (95% CI)	n	%	ROR (95% CI)	n	%	ROR (95% CI)	n	%	ROR (95% CI)
1	Constipation	2,887	17.93	10.32 (9.94,10.72)	495	2.96	3.60 (3.30,3.94)	159	2.70	2.86 (2.45,3.35)	31	2.85	1.77 (1.24,2.52)
2	Feeling abnormal	542	3.37	1.64 (1.51,1.79)	355	2.12	2.31 (2.08,2.57)	128	2.17	2.07 (1.74,2.46)	61	5.62	3.16 (2.45,4.06)
3	Throat tightness	46	0.29	1.36 (1.02,1.81)	32	0.19	2.02 (1.43,2.86)	29	0.49	4.57 (3.17,6.59)	13	1.20	6.53 (3.79,11.26)
4	Paraesthesia oral	38	0.24	2.08 (1.51,2.86)	16	0.10	1.87 (1.14,3.05)	11	0.19	3.19 (1.77,5.77)	9	0.83	8.34 (4.34,16.05)
5	Alopecia	1,164	7.23	3.31 (3.12,3.51)	582	3.48	3.53 (3.25,3.83)	182	3.09	2.73 (2.36,3.16)			
6	Anxiety	444	2.76%	1.12 (1.02,1.23)	378	2.26%	2.05 (1.86,2.27)	122	2.07%	1.64 (1.38,1.97)			
7	Weight increased	407	2.53	1.37 (1.24,1.51)	528	3.15	3.86 (3.54,4.20)	139	2.36	2.51 (2.12,2.96)			
8	Insomnia	386	2.40	1.19 (1.07,1.31)	203	1.21	1.34 (1.16,1.53)	93	1.58	1.52 (1.24,1.87)			
9	Myalgia	317	1.97	1.50 (1.34,1.68)	148	0.88	1.50 (1.27,1.76)	66	1.12	1.66 (1.31,2.12)			
10	Influenza like illness	274	1.70	2.96 (2.63,3.33)	75	0.45	1.72 (1.37,2.16)	34	0.58	1.94 (1.39,2.72)			
11	Hypoaesthesia	253	1.57	1.34 (1.19,1.52)	125	0.75	1.42 (1.19,1.69)	56	0.95	1.58 (1.22,2.06)			
12	Urticaria	253	1.57	1.14 (1.01,1.29)	296	1.77	2.88 (2.57,3.23)	132	2.24	3.19 (2.69,3.79)			
13	Paraesthesia	252	1.57	1.25 (1.11,1.42)	135	0.81	1.44 (1.21,1.70)	58	0.99	1.54 (1.19,1.99)			
14	Palpitations	245	1.52	1.67 (1.47,1.89)	120	0.72	1.75 (1.46,2.09)	85	1.44	3.09 (2.50,3.83)			
15	Abdominal distension	181	1.12	1.38 (1.19,1.59)	104	0.62	1.69 (1.40,2.05)	37	0.63	1.50 (1.08,2.07)			
16	Panic attack	85	0.53	1.99 (1.61,2.46)	57	0.34	2.85 (2.20,3.70)	34	0.58	4.23 (3.02,5.93)			
17	Fear of injection	66	0.41	6.07 (4.76,7.75)	56	0.33	11.02 (8.45,14.35)	12	0.20	5.79 (3.28,10.21)			
18	Menstruation irregular	53	0.33	3.32 (2.53,4.35)	29	0.17	3.87 (2.69,5.58)	9	0.15	2.98 (1.55,5.73)			
19	Abnormal dreams	52	0.32	2.32 (1.77,3.05)	29	0.17	2.77 (1.92,3.98)	10	0.17	2.37 (1.27,4.41)			
20	Raynaud’s phenomenon	50	0.31	8.28 (6.24,10.97)	35	0.21	12.31 (8.81,17.21)	14	0.24	12.12 (7.16,20.51)			
21	Muscle tightness	42	0.26	2.10 (1.55,2.84)	16	0.10	1.71 (1.04,2.79)	11	0.19	2.92 (1.62,5.28)			
22	Menstrual disorder	40	0.25	3.97 (2.90,5.42)	20	0.12	4.22 (2.72,6.56)	14	0.24	7.35 (4.35,12.43)			
23	Trichorrhexis	22	0.14	6.10 (3.99,9.30)	9	0.05	5.28 (2.74,10.17)	6	0.10	8.74 (3.92,19.51)			
24	Hormone level abnormal	15	0.09	2.20 (1.32,3.65)	13	0.08	4.08 (2.36,7.04)	5	0.08	3.89 (1.62,9.37)			
25	Oligomenorrhoea	7	0.04	3.73 (1.77,7.87)	4	0.02	4.55 (1.7,12.16)	3	0.05	8.48 (2.73,26.40)			
26	Concussion	36	0.22	3.32 (2.39,4.61)	12	0.07	2.35 (1.33,4.15)				6	0.55	9.34 (4.19,20.82)
27	Fibromyalgia	43	0.27	1.38 (1.02,1.86)	24	0.14	1.65 (1.10,2.46)				9	0.83	4.90 (2.55,9.43)
28	Blepharospasm	31	0.19	4.90 (3.44,6.99)				6	0.10	5.00 (2.24,11.14)	4	0.37	10.62 (3.98,28.33)
29	Pruritus				389	2.32	1.58 (1.43,1.74)	216	3.67	2.19 (1.91,2.50)	89	8.20	2.88 (2.34,3.55)
30	Pharyngeal swelling				22	0.13	2.20 (1.45,3.35)	12	0.20	2.99 (1.70,5.26)	8	0.74	6.35 (3.17,12.72)
31	Swollen tongue				34	0.20	2.02 (1.44,2.83)	26	0.44	3.84 (2.62,5.65)	6	0.55	2.82 (1.27,6.29)

Notes: AE: adverse event; ROR: reporting odd ratio; CI: confidence interval.

### 3.5 Injection-related AEs for the four anti-CGRP mAbs

Erenumab, fremanezumab, and galcanezumab are administered subcutaneously, so injection-related AEs were common. In contrast, eptinezumab is the only anti-CGRP mAb administered by intravenous infusion, so the corresponding adverse reaction is an infusion site reaction. Aggregating the AEs related to injection or infusion (Supplementary Table S5), 37 AEs related to injection and 8 AEs related to infusion were found. Specifically, the largest number of injection-site AEs associated with galcanezumab, amounting to 10,012 cases and accounting for 59.82% of all mined AEs; 6,099 cases of injection-site reactions associated with erenumab, accounting for 37.88%; 3,086 cases related to fremanezumab, accounting for 52.43%. 85 cases of infusion-related reactions have been reported with eptinezumab, which represents 7.83% of all AEs. Common injection site AEs included injection site pain, injection site erythema, injection site pruritus, and injection site haemorrhage.

## 4 Discussion

This study mined the AE signals of four anti-CGRP mAbs from the FAERS database using ROR and BCPNN. The ROR method has the advantages of simplicity of calculation, reduction of bias due to control group selection, and high sensitivity. However, the specificity is relatively low and prone to false positives. The BCPNN method, on the other hand, combines Bayesian logic and neural network structure for more stable results and higher specificity. Those two methods were combined in this study to reduce the results bias caused by a single algorithm. It is the first retrospective study to analyze and compare all post-marketing AEs related to the four drugs to date, intending to provide a reference for predicting AEs of anti-CGRP drugs and clinical drug selection.

The signal mining revealed that the primary SOC for the four antibodies was general disorders and administration site conditions, with injection site reactions being the most frequently reported AE, which was similar to the main AE described in the instruction. Nevertheless, the injection-related AEs observed in this study were more diverse, manifesting as injection site depression, swelling, bruising, urticaria, rash, warmth, induration, irritation, and extravasation, etc. The incidence of injection site reactions is high among FDA-approved self-injectable biologics, with up to 40% reported (Thomaidou and Ramot, 2019), which can directly reduce patient compliance and, thus, the drug’s efficacy. Nevertheless, the symptoms of injection-related reactions in this study were mild, and no medication discontinuation due to injection reactions has been reported. However, long-term subcutaneous drug administration may lead to fear of injection in patients. In this study, strong signals of fear of injection were mined for all three subcutaneously injected drugs, with erenumab (ROR = 6.07; 95% CI, 4.76–7.75), galcanezumab (ROR = 11.02; 95% CI, 8.45–14.35), fremanezumab (ROR = 5.79; 95% CI, 3.28–10.21). It is noteworthy that since eptinezumab is administered intravenously and at long intervals between doses, it has a low incidence of injection-induced AEs. Compared to the other three drugs, it may provide patients with a better treatment experience.

It can be seen from the reported AEs related to anti-CGRP mAbs that a large proportion of the cases were female patients, consistent with the epidemiological profile of migraine (Broner et al., 2017; Charles, 2017). Females are more likely to suffer migraine attacks than males, with hormonal fluctuations, particularly changes in estrogen levels, playing an important role (Broner et al., 2017). Migraine attacks in women are more frequent, severe, and prolonged and are accompanied by many symptoms, such as photophobia, phonophobia, and nausea (Boardman et al., 2003; Pavlovic et al., 2017). The AEs of hormone level abnormal, menstrual disorder, menstruation irregular, and oligomenorrhoea were mined in the three subcutaneously injected drugs in this study, which had not been reported previously in previous studies. A decrease in estrogen during the luteal phase of the menstrual cycle is an important trigger for migraine attacks during menstruation, and 70% of women with migraine can develop menstrual migraines (Calhoun, 2018). This might be due to the change of CGRP levels during the menstrual cycle (Raffaelli et al., 2021), while CGRP can promote neurogenic inflammation of the endometrial tissues (Yan et al., 2019). Therefore, blocking CGRP could induce changes in menstruation. However, there were no relevant signals of menstrual disorder have been mined in the novel drug for eptinezumab.

Constipation was the most reported post-marketing AE of erenumab. It had been reported in previous clinical trials of erenumab (Sun et al., 2016; Tepper et al., 2017; Takeshima et al., 2021) and mentioned in real-world studies (Ornello et al., 2020; Deligianni et al., 2021). Compared with other drugs, erenumab showed a stronger signal of constipation (ROR = 10.32, 95% CI, 9.94–10.72) and was reported most frequently. However, there was no mention of constipation in any of the clinical trials of galcanezumab, fremanezumab, or eptinezumab (Dodick et al., 2018; Skljarevski et al., 2018; Ferrari et al., 2019; Lipton et al., 2020; Mulleners et al., 2020), but the signal was strong in our study. According to another study based on the FAERS database of three drugs administered subcutaneously 6 months after marketing, only erenumab was reported to cause constipation (Silberstein et al., 2023). This discrepancy between the present study and the previous one may be because short-term constipation may not be easily taken seriously by patients, and only chronic constipation caused by long-term medication may attract their attention. CGRP, as an endogenous neuropeptide, is also distributed in the primary afferent nerve cells of the submucosal plexus of the enteric nervous system; it transmits signals from various physical and chemical stimuli in the intestinal lumen or intestinal wall and is involved in regulating the functions and activities of the gastrointestinal tract (Clifton et al., 2007; Holzer and Holzer-Petsche, 2021). Hence, constipation during treatment with anti-CGRP mAb treatment has a biological basis. There has been a warning included in the instructions for erenumab that the drug may cause constipation companied serious complications. It is the first time that eptinezumab has been reported to cause constipation, and the signal strength is (ROR = 1.77, 95% CI, 1.24–2.52) with 31 cases.

Alopecia was another most common AE, observed in three subcutaneous injections, which was only reported post-marketing (Ruiz et al., 2023) and not found in the clinical trial phase. Initially, the reports did not attract clinical attention and were not sufficient for meaningful analysis due to the small number of cases at the time. Nevertheless, a more recent study has observed an association between CGRP inhibitor use and alopecia in migraine sufferers (Woods, 2022). Likewise, erenumab, fremanezumab, and galcanezumab all had strong signals of alopecia in our study, with signal intensities of 3.31 (95% CI, 3.12–3.51), 2.73 (95% CI, 2.36–3.16), and 3.53 (95% CI, 3.25–3.83), respectively. Thus, it is essential to focus on the long-term AE of alopecia in clinical practice, since alopecia may affect patients’ quality of life (Tzur et al., 2022). Moreover, the AE of trichorrhexis which may be associated with alopecia was tapped in those three drugs. CGRP plays an important role in maintaining the immune privilege of hair follicles (Pi et al., 2013). In addition, reduced levels of CGRP result in reduced blood supply to the hair follicle (Rossi et al., 1997). The repetitive activation of C fibers in migraine can also result in the depletion of substance P and CGRP, leading to the loss of hair growth promotion and reduction of microvascular blood flow to the hair follicle (Bedrin and Dougherty, 2020). Therefore, drugs inhibiting CGRP can lead to alopecia. In the reports of eptinezumab, we temporarily did not observe a signal related to alopecia.

A new signal that was not mentioned in the instructions of any anti-CGRP mAbs but had a strong signal was found in the present study. Raynaud phenomenon is an exaggerated physiological response to cold exposure or emotional stress characterized by a triphasic color change in extremities due to impaired blood circulation that can lead to ulceration, scarring, or gangrene (Goundry et al., 2012). There have been some previous case reports reported that fremanezumab, galcanezumab, and erenumab could induce Raynaud’s phenomenon (Evans, 2019; Manickam et al., 2021), but the number was less. In this study, the cases were reported more frequently and with a strong signal for all three drugs: ROR = 8.26 (95% CI, 6.24–10.97) for erenumab, ROR = 12.31 (95% CI, 8.81–17.21) for galcanezumab, and ROR = 12.12 (95% CI, 7.16–20.51) for fremanezumab. CGRP is stored in vesicles on sensory nerve endings, and activation of its receptors contributes to blood vessel dilation. One study showed that CGRP immunoreactive fibers were significantly reduced in the epidermis and subepidermis of skin in patients with the Raynaud phenomenon compared to controls (Terenghi et al., 1991), suggested that blocking CGRP could cause the Raynaud phenomenon. Therefore, while anti-CGRP drugs can reduce the release of CGRP and relieve migraine attacks, they also can induce Raynaud’s phenomenon in some cases. A real-world study showed that anti-CGRP drugs could induce or aggravate Raynaud’s phenomenon with a significantly stronger signal than triphenylamine, which is a migraine drug that can induce the Raynaud phenomenon (Gerard et al., 2022). Although the Raynaud phenomenon was rare and the use of anti-CGRP mAbs in patients with the Raynaud phenomenon had a low incidence of microvascular complications, this was still considered worthy of attention in clinical practice (Breen et al., 2021).

The AE of weight increased have been reported frequently for all three drugs administered subcutaneously, with the number of 407, 528, and 139 for erenumab, galcanezumab, and fremanezumab, respectively. To date, there have been no case reports of anti-CGRP mAbs causing weight increase. CGRP and amylin are both members of the same peptide family and have been investigated as potential treatments for metabolic diseases (Sonne et al., 2021). The release of CGRP may play an important role in adipocyte lipid metabolism and thus in systemic metabolism (Nogueiras et al., 2010). Anti-CGRP mAbs may inhibit the release of CGRP thereby affecting metabolism and leading to weight increase. An explorative, prospective, questionnaire-based study showed that 18.8% reported an increase in body weight 3 months after treatment with anti-CGRP mAbs (Iannone et al., 2022). The severity of the weight increase caused by anti-CGRP drugs is unknown based on current reports, but for patients who need or are undergoing weight control, the three subcutaneously administered drugs can induces the risk of weight control failure.

Migraine is the second most common neurological disorder in which the patient has prodromal or concomitant symptoms during the attack. In our study, we have discovered signals that may be associated with co-morbidities or concomitant symptoms and presented in at least three drugs, including feeling abnormal, anxiety, insomnia, hypoaesthesia, paraesthesia, palpitations, panic attack, etc. In addition, this study also uncovered musculoskeletal and connective tissue disorders, such as muscle tightness, muscle spasm, myalgia, and fibromyalgia, which were reported in the erenumab but not in the instruction of other drugs. However, in this study there was no detection of hypertension-related signals. Since CGRP is a microvessel dilator, vascular-related adverse effects have been monitored since the beginning of the clinical trials. This study detected cardiovascular signals, including palpitations, postural tachycardia syndromes, and coronary artery spasms, while no hypertension signals were found. Recently, Sessa et al. showed no significant association between CGRP receptor antagonists and increased risks of hypertension events, consistent with the present study (Sessa and Andersen, 2021). Further data will be required at a later stage to continue to detect the relevant AEs.

Eptinezumab is a newly marketed anti-CGRP mAb that is administered intravenously once every 3 months, which greatly improves patient compliance, especially with patients who fear injections. Previous studies have demonstrated a favorable safety and tolerability for eptinezumab in adult patients with migraine. According to this study, the AEs of post-marketing with high incidence observed in the three subcutaneous anti-CGRP mAbs, including alopecia, weight gain, urticaria, and Raynaud’s phenomenon, were not observed in eptinezumab. It may be more beneficial to choose eptinezumab for patients suffering from previous allergic conditions, afraid of alopecia, with a history of Raynaud’s phenomenon, and worried about obesity. Furthermore, no reports of menstrual disorders, menstruation irregular, and oligomenorrhoea were reported with eptinezumab, and this drug may be more suitable for women of childbearing age. The common AEs to eptinezumab are fatigue, throat irritation, and pruritus. It is possible that fatigue is a concomitant symptom of migraine, which is relieved during the course of the drug therapy (usually 4 weeks after the second dose) (Lipton et al., 2021). Pruritus is a common allergic skin reaction and is labeled in the instructions, which was observed in fremanezumab and galcanezumab. Throat irritation (ROR = 29.51; 95% CI, 24.15–36.06) was a new and stronger signal AE that should receive attention. There are also several signals associated with throat irritation, including oropharyngeal pain, throat tightness, and swelling of the pharynx. An early study suggested autonomic and peptidergic innervation in the human larynx (Hauser-Kronberger et al., 1993) and that the concentration of pharyngeal sensory CGRP positively correlated with pharyngeal function (Tomsen et al., 2022). Therefore, peptinezumab should be avoided in patients with laryngeal disorders. Of note, due to the late launch of eptinezumab, certain adverse effects may not have yet been reported. Consequently, it is imperative to maintain ongoing surveillance of the AEs linked to anti-GCRP mAbs.

There are several limitations to this study. First, spontaneous reporting is prone to reporting bias, such as incomplete data, duplicate data, unstandardized completion, and high variability in data quality. Second, FDA does not require that a causal relationship between a product and event be proven, and reports do not always contain enough detail to properly evaluate an event. The reports in FAERS submitted may not fully reflect the causal relationship between exposure and the outcome, and it is impossible to use such data to determine the incidence of a particular reaction in a population. Therefore, additional studies are needed to determine causality. Third, the AE reporting of new drugs can suffer from the Weber effect, in which higher rates of AEs are reported in the early period of drug approval (Arora et al., 2017). Nevertheless, the Weber effect has not been observed in FAERS (Hoffman et al., 2014; Arora et al., 2017). Fourth, in this study, many signals related to the indication of migraine were mined, such as migraine with aura, tension migraine, vestibular migraine, migraine from drug overuse, headache, and post-traumatic headache. And concomitant symptoms associated with migraine attack such as fatigue, poor concentration, anxiety, irritability, irritability, tearing, photophobia, phonophobia, vertigo, dizziness, neck pain, etc. Since these mined signals are associated with the indication of the anti-CGRP mAbs or symptoms accompanied by migraine, it is impossible to determine whether the drugs caused them or whether the drugs exacerbated the symptoms. Fifth, considering the relatively brief duration that these drugs, particularly eptinezumab, have been available on the market, it is essential to maintain continuous monitoring of their safety.

## 5 Conclusion

This study conducted a thorough analysis and comparison of post-marketing AE signals associated with four anti-CGRP mAbs which contribute to understanding the safety profile of anti-CGRP mAbs in clinical practice, providing valuable insights for clinical drug selection.

## Data Availability

The original contributions presented in the study are included in the article/Supplementary Materials, further inquiries can be directed to the corresponding author.
