# Supplementary material for: Adverse event reporting of four anti-Calcitonin gene-related peptide monoclonal antibodies for migraine prevention: a real-world study based on the FDA adverse event reporting system
Source: Front Pharmacol. 2024 Jan 9;14:1257282. doi: 10.3389/fphar.2023.1257282 (PMC10803415; doi:10.3389/fphar.2023.1257282)
Supplement: Supplementary file 1 [file Table1.docx]

**Supplementary Table S1.** PT signal detection under each SOC for Erenumab

| SOC | PT | n | % | ROR | IC |
| --- | --- | --- | --- | --- | --- |
| General disorders and administration site conditions | Injection site pain | 2267 | 14.08 | 6.86(6.57,7.15) | 2.71(2.65,2.77) |
|  | Injection site haemorrhage | 926 | 5.75 | 9.23(8.65,9.86) | 3.14(3.04,3.23) |
|  | Feeling abnormal | 542 | 3.37 | 1.64(1.51,1.79) | 0.71(0.58,0.83) |
|  | Injection site bruising | 535 | 3.32 | 5.96(5.47,6.49) | 2.53(2.40,2.65) |
|  | Injection site erythema | 437 | 2.71 | 4.05(3.68,4.45) | 1.98(1.85,2.12) |
|  | Injection site swelling | 418 | 2.60 | 5.32(4.83,5.86) | 2.37(2.23,2.51) |
|  | Influenza like illness | 274 | 1.70 | 2.96(2.63,3.33) | 1.54(1.37,1.72) |
|  | Injection site reaction | 257 | 1.60 | 3.40(3.01,3.85) | 1.74(1.56,1.92) |
|  | Injection site pruritus | 215 | 1.34 | 3.26(2.85,3.73) | 1.68(1.48,1.87) |
|  | Injection site urticaria | 158 | 0.98 | 5.89(5.03,6.90) | 2.49(2.26,2.72) |
|  | Injection site rash | 134 | 0.83 | 4.16(3.51,4.93) | 2.00(1.75,2.26) |
|  | Injection site mass | 120 | 0.75 | 2.03(1.70,2.43) | 1.00(0.74,1.27) |
|  | Injection site extravasation | 109 | 0.68 | 5.62(4.65,6.80) | 2.41(2.13,2.68) |
|  | Injection site indentation | 107 | 0.66 | 29.77(24.37,36.36) | 4.43(4.14,4.73) |
|  | Injection site discomfort | 70 | 0.43 | 5.09(4.02,6.45) | 2.25(1.90,2.59) |
|  | Injection site warmth | 67 | 0.42 | 4.21(3.31,5.36) | 1.99(1.64,2.34) |
|  | Injection site discolouration | 57 | 0.35 | 4.06(3.12,5.27) | 1.93(1.55,2.31) |
|  | Injection site irritation | 39 | 0.24 | 4.51(3.28,6.19) | 2.03(1.57,2.49) |
|  | Injection site injury | 29 | 0.18 | 4.16(2.88,6.00) | 1.89(1.36,2.43) |
|  | Injection site vesicles | 28 | 0.17 | 4.13(2.84,6.00) | 1.88(1.34,2.42) |
|  | Injection site induration | 22 | 0.14 | 1.61(1.06,2.45) | 0.69(0.04,1.25) |
|  | Fibrosis | 20 | 0.12 | 5.65(3.63,8.80) | 2.19(1.55,2.82) |
|  | Injection site hypoaesthesia | 18 | 0.11 | 8.68(5.43,13.89) | 2.60(1.93,3.27) |
|  | Injection site hypersensitivity | 14 | 0.09 | 3.61(2.13,6.11) | 1.61(0.86,2.36) |
|  | Injection site papule | 13 | 0.08 | 2.33(1.35,4.03) | 1.08(0.31,1.86) |
|  | Injection site inflammation | 12 | 0.07 | 2.09(1.18,3.68) | 0.94(0.14,1.74) |
|  | Injection site paraesthesia | 10 | 0.06 | 5.42(2.90,10.14) | 1.93(1.05,2.81) |
|  | Injection site scar | 9 | 0.06 | 2.31(1.20,4.45) | 1.02(0.11,1.94) |
|  | Injection site macule | 6 | 0.04 | 12.23(5.39,27.72) | 2.21(1.09,3.33) |
|  | Injection site coldness | 5 | 0.03 | 7.92(3.25,19.28) | 1.86(0.66,3.06) |
| Gastrointestinal disorders | Constipation | 2887 | 17.93 | 10.32(9.94,10.72) | 3.27(3.22,3.33) |
|  | Abdominal distension | 181 | 1.12 | 1.38(1.19,1.59) | 0.46(0.24,0.67) |
|  | Irritable bowel syndrome | 70 | 0.43 | 2.59(2.05,3.28) | 1.33(0.99,1.68) |
|  | Impaired gastric emptying | 40 | 0.25 | 3.76(2.75,5.14) | 1.80(1.35,2.26) |
|  | Paraesthesia oral | 38 | 0.24 | 2.08(1.51,2.86) | 1.01(0.54,1.47) |
|  | Hypoaesthesia oral | 33 | 0.20 | 1.86(1.32,2.62) | 0.89(0.36,1.35) |
|  | Gastrointestinal pain | 31 | 0.19 | 2.01(1.41,2.87) | 0.96(0.45,1.47) |
|  | Faecaloma | 29 | 0.18 | 4.11(2.85,5.94) | 1.88(1.35,2.41) |
|  | Bowel movement irregularity | 23 | 0.14 | 1.88(1.25,2.83) | 1.58(0.87,2.06) |
|  | Gastrointestinal motility disorder | 23 | 0.14 | 3.01(2.00,4.55) | 1.46(0.87,2.06) |
|  | Colitis ischaemic | 18 | 0.11 | 2.35(1.48,3.74) | 1.13(0.46,1.79) |
|  | Dyschezia | 14 | 0.09 | 2.40(1.42,4.07) | 1.13(0.38,1.88) |
|  | Ileus paralytic | 13 | 0.08 | 2.84(1.64,4.91) | 1.32(0.54,2.09) |
|  | Gastrointestinal hypomotility | 11 | 0.07 | 7.67(4.21,13.97) | 2.28(1.43,3.12) |
|  | Gastrointestinal obstruction | 10 | 0.06 | 2.62(1.41,4.89) | 1.18(0.31,2.06) |
|  | Intestinal ulcer | 7 | 0.04 | 3.39(1.61,7.14) | 1.37(0.35,2.40) |
|  | Oesophageal spasm | 6 | 0.04 | 3.23(1.44,7.23) | 1.28(0.19,2.38) |
| Skin and subcutaneous tissue disorders | Alopecia | 1164 | 7.23 | 3.31(3.12,3.51) | 1.70(1.61,1.78) |
|  | Urticaria | 253 | 1.57 | 1.14(1.00,1.29) | 0.19(0.01,0.37) |
|  | Trichorrhexis | 22 | 0.14 | 6.10(3.99,9.30) | 2.30(1.69,2.90) |
|  | Rosacea | 14 | 0.09 | 2.10(1.24,3.55) | 0.96(0.21,1.71) |
|  | Skin oedema | 6 | 0.04 | 5.22(2.33,11.72) | 1.69(0.59,2.79) |
| Psychiatric disorders | Anxiety | 444 | 2.76 | 1.12(1.02,1.23) | 0.16(0.03,0.30) |
|  | Insomnia | 386 | 2.40 | 1.19(1.07,1.31) | 0.24(0.10,0.39) |
|  | Depression | 338 | 2.10 | 1.26(1.13,1.40) | 0.33(0.17,0.48) |
|  | Panic attack | 85 | 0.53 | 1.99(1.61,2.46) | 0.98(0.66,1.28) |
|  | Fear of injection | 66 | 0.41 | 6.07(4.76,7.75) | 2.47(2.11,2.83) |
|  | Sleep disorder due to a general medical condition | 61 | 0.38 | 2.32(1.77,3.05) | 1.17(0.77,1.57) |
|  | Abnormal dreams | 52 | 0.32 | 2.32(1.77,3.05) | 1.21(0.77,1.7) |
|  | Panic disorder | 12 | 0.07 | 2.49(1.41,4.40) | 1.31(0.35,1.96) |
| Musculoskeletal and connective tissue disorders | Muscle spasms | 625 | 3.88 | 2.69(2.49,2.91) | 1.41(1.29,1.52) |
|  | Myalgia | 317 | 1.97 | 1.50(1.34,1.68) | 0.58(0.42,0.75) |
|  | Muscle twitching | 52 | 0.32 | 1.96((1.49,2.58) | 0.97(0.54,1.34) |
|  | Fibromyalgia | 43 | 0.27 | 1.38(1.02,1.86) | 0.46(0.01,0.88) |
|  | Muscle tightness | 42 | 0.26 | 2.10(1.55,2.84) | 1.03(0.58,1.47) |
|  | Temporomandibular joint syndrome | 8 | 0.05 | 2.31(1.15,4.63) | 1.00(0.04,1.97) |
|  | Collagen disorder | 4 | 0.03 | 5.31(1.97,14.29) | 1.50(0.19,2.80) |
| Nervous system disorders | Hypoaesthesia | 253 | 1.57 | 1.34(1.19,1.52） | 0.42(0.24,0.60） |
|  | Paraesthesia | 252 | 1.57 | 1.25(1.11,1.42) | 0.32(0.14,0.50) |
|  | Aphasia | 57 | 0.35 | 1.47(1.14,1.91) | 0.56(0.16,0.92) |
|  | Reversible cerebral vasoconstriction syndrome | 16 | 0.10 | 4.44(2.71,7.28) | 1.87(1.16,2.58) |
|  | Sleep deficit | 12 | 0.07 | 2.99(1.69,5.28) | 1.36(0.56,2.17) |
|  | Psychogenic seizure | 9 | 0.06 | 5.37(2.77,10.38) | 1.88(0.96,2.81) |
|  | Cerebrospinal fluid leakage | 7 | 0.04 | 2.71(1.29,5.72) | 1.15(0.13,2.18) |
|  | Transient global amnesia | 4 | 0.03 | 4.65(1.73,12.51) | 1.41(0.11,2.72) |
|  | Meningeal disorder | 3 | 0.02 | 7.73(2.45,24.37) | 1.51(0.04,2.98) |
|  | Pineal gland cyst | 3 | 0.02 | 23.20(7.12,75.53) | 1.81(0.29,3.32) |
| Investigations | Weight increased | 407 | 2.53 | 1.37(1.24,1.51) | 0.45(0.31,0.60) |
|  | Hormone level abnormal | 15 | 0.09 | 2.20(1.32,3.65) | 1.13(0.30,1.75) |
|  | Histone antibody positive | 3 | 0.02 | 19.24(5.96,62.12) | 4.17(0.27,3.28) |
| Cardiac disorders | Palpitations | 245 | 1.52 | 1.67(1.47,1.89) | 0.73(0.55,0.91) |
|  | Postural orthostatic tachycardia syndrome | 15 | 0.09 | 9.21(5.51,15.42) | 2.58(1.84,3.31) |
|  | Coronary artery dissection | 7 | 0.04 | 5.32(2.52,11.24) | 1.77(0.74,2.80) |
| Reproductive system and breast disorders | Menstruation irregular | 53 | 0.33 | 3.32(2.53,4.35) | 1.66(1.26,2.05) |
|  | Menstrual disorder | 40 | 0.25 | 3.97(2.90,5.42) | 1.87(1.42,2.33) |
|  | Amenorrhoea | 37 | 0.23 | 2.39(1.73,3.30) | 1.20(0.73,1.67) |
|  | Intermenstrual bleeding | 26 | 0.16 | 1.72(1.17,2.53） | 0.78(0.18,1.30) |
|  | Postmenopausal haemorrhage | 8 | 0.05 | 2.60(1.29,5.21) | 1.13(0.17,2.10) |
|  | Premature menopause | 7 | 0.04 | 7.36(3.47,15.6) | 2.02(0.98,3.05) |
|  | Oligomenorrhoea | 7 | 0.04 | 3.73(1.77,7.87) | 1.46(0.44,2.49) |
|  | Ovarian disorder | 4 | 0.03 | 4.31(1.60,11.58) | 1.36(0.06,2.67) |
| Injury, poisoning and procedural complications | Concussion | 36 | 0.22 | 3.32(2.39,4.61) | 1.63(1.15,2.11) |
|  | Injection related reaction | 17 | 0.11 | 6.01(3.71,9.72) | 2.21(1.52,2.90) |
|  | Lack of injection site rotation | 16 | 0.10 | 6.44(3.92,10.58) | 2.26(1.56,2.97) |
|  | Craniocerebral injury | 12 | 0.08 | 2.61(1.48,4.61) | 1.21(0.40,2.01) |
|  | Post-traumatic neck syndrome | 6 | 0.04 | 5.12(2.28,11.49) | 1.67(0.57,2.78) |
|  | Post concussion syndrome | 4 | 0.03 | 8.69(3.21,23.53) | 1.76(0.44,3.07) |
| Vascular disorders | Raynaud's phenomenon | 50 | 0.31 | 8.28(6.24,10.97) | 2.82(2.41,3.23) |
|  | Hypertensive urgency | 4 | 0.03 | 4.35(1.62,11.67) | 1.37(0.07,2.67) |
| Respiratory, thoracic and mediastinal disorders | Throat tightness | 46 | 0.29 | 1.36(1.02,1.81) | 0.01(0.85,0.44) |
| Eye disorders | Blepharospasm | 31 | 0.19 | 4.90(3.44,6.99) | 2.11(1.59,2.62) |
|  | Saccadic eye movement | 3 | 0.02 | 10.66(3.36,33.8) | 3.36(0.15,3.11) |
| Immune system disorders | Autoimmune disorder | 21 | 0.13 | 1.58(1.03,2.43) | 0.66(0.00,1.24) |
| Endocrine disorders | Autoimmune thyroiditis | 11 | 0.07 | 1.95(1.08,3.52) | 0.96(0.11,1.68) |
| Renal and urinary disorders | Cystitis interstitial | 6 | 0.04 | 3.10(1.39,6.93) | 1.24(0.15,2.34) |
| Total |  | 16099 | 100 |  |  |

Note：SOC: System Organ Class; PT: preferred term; ROR: reporting odd ratio; IC: information components
