# Supplementary material for: Adverse event reporting of four anti-Calcitonin gene-related peptide monoclonal antibodies for migraine prevention: a real-world study based on the FDA adverse event reporting system
Source: Front Pharmacol. 2024 Jan 9;14:1257282. doi: 10.3389/fphar.2023.1257282 (PMC10803415; doi:10.3389/fphar.2023.1257282)
Supplement: Supplementary file 2 [file Table2.docx]

**Supplementary Table S2.** PT signal detection under each SOC for Galcanezumab

| SOC | PT | N | % | ROR | IC |
| --- | --- | --- | --- | --- | --- |
| General disorders and administration site conditions | Injection site pain | 4,079 | 24.37 | 29.22(28.27,30.2） | 4.65(4.60,4.70） |
|  | Injection site erythema | 896 | 5.35 | 18.39(17.2,19.67） | 4.10(4.00,4.20） |
|  | Injection site haemorrhage | 831 | 4.97 | 17.89(16.68,19.18） | 4.06(3.96,4.17） |
|  | Injection site pruritus | 676 | 4.04 | 22.92(21.21,24.76） | 4.40(4.28,4.51） |
|  | Injection site swelling | 663 | 3.96 | 18.53(17.14,20.03） | 4.11(4.00,4.22） |
|  | Injection site reaction | 595 | 3.56 | 17.39(16.02,18.88） | 4.02(3.90,4.14） |
|  | Injection site bruising | 434 | 2.59 | 10.37(9.42,11.4） | 3.31(3.17,3.45） |
|  | Injection site urticaria | 378 | 2.26 | 31.43(28.32,34.87） | 4.78(4.63,4.93） |
|  | Feeling abnormal | 355 | 2.12 | 2.31(2.08,2.57） | 1.19(1.04,1.35） |
|  | Injection site mass | 346 | 2.07 | 12.83(11.53,14.28） | 3.59(3.44,3.75） |
|  | Injection site rash | 270 | 1.61 | 18.36(16.26,20.74） | 4.06(3.88,4.24） |
|  | Injection site warmth | 177 | 1.06 | 24.61(21.17,28.62） | 4.38(4.16,4.61） |
|  | Injection site discomfort | 112 | 0.67 | 17.72(14.68,21.39） | 3.91(3.63,4.18） |
|  | Injection site injury | 104 | 0.62 | 33.40(27.4,40.71） | 4.61(4.32,4.90） |
|  | Injection site induration | 83 | 0.50 | 13.29(10.69,16.53） | 3.51(3.19,3.82） |
|  | Influenza like illness | 75 | 0.45 | 1.72(1.37,2.18) | 0.78(0.44,1.10) |
|  | Injection site irritation | 69 | 0.41 | 17.36(13.66,22.06） | 3.78(3.43,4.13） |
|  | Feeling hot | 62 | 0.37 | 1.70(1.32,2.18) | 0.76(0.38,1.11) |
|  | Swelling face | 54 | 0.32 | 1.38(1.06,1.81) | 0.47(0.07,0.85) |
|  | Injection site discolouration | 50 | 0.30 | 7.62(5.76,10.08） | 2.74(2.33,3.14） |
|  | Injection site hypersensitivity | 42 | 0.25 | 23.87(17.53,32.51） | 3.92(3.47,4.37） |
|  | Injection site inflammation | 34 | 0.20 | 12.88(9.17,18.1） | 3.24(2.75,3.74） |
|  | Injection site vesicles | 32 | 0.19 | 10.15(7.16,14.41） | 2.97(2.46,3.48） |
|  | Injection site extravasation | 23 | 0.14 | 2.5(1.66,3.77） | 1.23(0.64,1.82） |
|  | Injection site scar | 20 | 0.12 | 11.13(7.15,17.32） | 2.89(2.25,3.53） |
|  | Injection site haematoma | 16 | 0.10 | 7.14(4.36,11.69） | 2.38(1.67,3.08） |
|  | Injection site nodule | 15 | 0.09 | 2.7(1.63,4.49） | 1.28(0.56,2.01） |
|  | Injection site papule | 11 | 0.07 | 4.23(2.34,7.66） | 1.73(0.89,2.57） |
|  | Injection site paraesthesia | 11 | 0.07 | 12.82(7.05,23.3） | 2.68(1.83,3.52） |
|  | Injection site macule | 8 | 0.05 | 35.52(17.39,72.58） | 2.86(1.87,3.85） |
|  | Injection site laceration | 4 | 0.02 | 13.04(4.84,35.13） | 1.93(0.62,3.24） |
|  | Injection site oedema | 4 | 0.02 | 4.15(1.55,11.11） | 1.34(0.05,2.64） |
|  | Injection site hypoaesthesia | 4 | 002 | 4.03(1.51,10.79） | 1.32(0.03,2.62） |
|  | Injection site coldness | 3 | 0.02 | 10.07(3.21,31.54） | 1.62(0.16,3.08） |
|  | Injection site streaking | 3 | 0.02 | 28.19(8.84,89.9） | 1.85(0.36,3.33） |
| Skin and subcutaneous tissue disorders | Alopecia | 582 | 3.48 | 3.53(3.25,3.83） | 1.79(1.67,1.91） |
|  | Pruritus | 389 | 2.32 | 1.58(1.43,1.74) | 0.65(0.50,0.79) |
|  | Rash | 381 | 2.28 | 1.27(1.15,1.40) | 0.34(0.19,0.49) |
|  | Urticaria | 296 | 1.77 | 2.88(2.57,3.23） | 1.50(1.34,1.67） |
|  | Rash pruritic | 44 | 0.26 | 1.38(1.02,1.85) | 0.46(0.02,0.88) |
|  | Trichorrhexis | 9 | 0.05 | 5.28(2.74,10.17） | 1.88(0.96,2.8） |
| Psychiatric disorders | Anxiety | 378 | 2.26 | 2.05(1.86,2.27） | 1.02(0.88,1.17） |
|  | Insomnia | 203 | 1.21 | 1.34(1.16,1.53) | 0.41(0.21,0.61) |
|  | Depression | 187 | 1.12 | 1.49(1.29,1.72) | 0.57(0.36,0.78) |
|  | Stress | 120 | 0.72 | 2.51(2.09,3.00） | 1.30(1.04,1.56） |
|  | Nervousness | 63 | 0.38 | 2.22(1.74,2.85） | 1.12(0.76,1.48） |
|  | Panic attack | 57 | 0.34 | 2.85(2.20,3.70） | 1.46(1.08,1.84） |
|  | Fear of injection | 56 | 0.33 | 11.02(8.45,14.35） | 3.20(2.82,3.59） |
|  | Abnormal dreams | 29 | 0.17 | 2.77(1.92,3.98） | 1.38(0.85,1.91） |
|  | Thinking abnormal | 26 | 0.16 | 2.55(1.73,3.74） | 1.26(0.71,1.82） |
|  | Panic reaction | 23 | 0.14 | 5.68(3.76,8.56） | 2.24(1.65,2.83） |
|  | Sleep disorder due to general medical condition, insomnia type | 11 | 0.07 | 3.07(1.7,5.55） | 1.38(0.55,2.22） |
|  | Distractibility | 4 | 0.02 | 5.9(2.2,15.81） | 1.57(0.27,2.87） |
|  | Dissociative disorder | 3 | 0.02 | 7.49(2.4,23.38） | 1.51(0.05,2.96） |
| Gastrointestinal disorders | Constipation | 495 | 2.96 | 3.60(3.30,3.94） | 1.82(1.69,1.95） |
|  | Abdominal distension | 104 | 0.62 | 1.69(1.40,2.05) | 0.76(0.46,1.03) |
|  | Swollen tongue | 34 | 0.20 | 2.02(1.44,2.83） | 0.97(0.48,1.46） |
|  | Lip swelling | 30 | 0.18 | 1.54(1.08,2.21) | 0.63(0.08,1.12) |
|  | Paraesthesia oral | 16 | 0.10 | 1.87(1.14,3.05) | 0.90(0.12,1.53) |
|  | Tongue haemorrhage | 4 | 0.02 | 6.21(2.32,16.65） | 1.60(0.30,2.90） |
| Nervous system disorders | Paraesthesia | 135 | 0.81 | 1.44(1.21,1.70) | 0.52(0.27,0.76) |
|  | Hypoaesthesia | 125 | 0.75 | 1.42(1.19,1.69) | 0.50(0.24,0.76) |
|  | Memory impairment | 125 | 0.75 | 1.35(1.13,1.61) | 0.43(0.17,0.68) |
|  | Burning sensation | 57 | 0.34 | 1.37(1.06,1.78) | 0.46(0.07,0.83) |
|  | Speech disorder | 48 | 0.29 | 1.54(1.16,2.05) | 0.63(0.20,1.02) |
|  | Disturbance in attention | 45 | 0.27 | 1.44(1.08,1.93) | 0.53(0.09,0.94) |
|  | Mental impairment | 36 | 0.22 | 2.52(1.82,3.50） | 1.27(0.80,1.75） |
|  | Cerebral disorder | 16 | 0.10 | 2.63(1.61,4.3） | 1.26(0.56,1.96） |
|  | Allodynia | 6 | 0.04 | 12.39(5.52,27.83） | 2.23(1.12,3.33） |
|  | Vertebrobasilar artery dissection | 4 | 0.02 | 19.96(7.36,54.11） | 2.05(0.73,3.37） |
| Musculoskeletal and connective tissue disorders | Arthralgia | 335 | 2.00 | 1.23(1.10,1..37) | 0.29(0.13,0.45) |
|  | Myalgia | 148 | 0.88 | 1.50(1.27,1.76) | 0.58(0.34,0.81) |
|  | Fibromyalgia | 24 | 0.14 | 1.65(1.10,2.46) | 0.72(0.10,1.26) |
|  | Musculoskeletal discomfort | 24 | 0.14 | 2.10(1.41,3.14） | 1.01(0.43,1.58） |
|  | Muscle twitching | 20 | 0.12 | 1.61(1.04,2.50) | 0.68(0.01,1.41) |
|  | Muscle tightness | 16 | 0.10 | 1.71(1.04,2.79) | 0.77(0.01,1.41) |
| Investigations | Weight increased | 528 | 3.15 | 3.86(3.54,4.20） | 1.92(1.79,2.05） |
|  | Hormone level abnormal | 13 | 0.08 | 4.08(2.36,7.04） | 1.73(0.96,2.51） |
|  | Body temperature fluctuation | 6 | 0.04 | 3.3(1.48,7.37） | 1.31(0.21,2.4） |
| Immune system disorders | Hypersensitivity | 261 | 1.56 | 2.00(1.77,2.26） | 0.99(0.81,1.17） |
|  | Anaphylactic reaction | 51 | 0.30 | 1.51(1.15,1.99) | 0.59(0.18,0.98) |
|  | Immune system disorder | 17 | 0.10 | 1.83(1.13,2.94) | 0.87(0.12,1.48) |
| Eye disorders | Visual impairment | 194 | 1.16 | 2.23(1.94,2.57） | 1.14(0.93,1.35） |
| Reproductive system and breast disorders | Menstruation irregular | 29 | 0.17 | 3.87(2.69,5.58） | 1.81(1.28,2.34） |
|  | Heavy menstrual bleeding | 23 | 0.14 | 1.63(1.08,2.45) | 0.70(0.07,1.25) |
|  | Menstrual disorder | 20 | 0.12 | 4.22(2.72,6.56） | 1.86(1.23,2.5） |
|  | Menstruation delayed | 18 | 0.11 | 3.44(2.16,5.46） | 1.6(0.94,2.26） |
|  | Amenorrhoea | 16 | 0.10 | 2.21(1.35,3.6） | 1.04(0.34,1.74） |
|  | Intermenstrual bleeding | 15 | 0.09 | 2.12(1.28,3.52） | 0.98(0.26,1.71） |
|  | Polymenorrhoea | 6 | 0.04 | 3.78(1.69,8.44） | 1.43(0.34,2.53） |
|  | Oligomenorrhoea | 4 | 0.02 | 4.55(1.7,12.16） | 1.41(0.11,2.7） |
| Cardiac disorders | Palpitations | 120 | 0.72 | 1.75(1.46,2.09) | 0.80(0.53,1.06) |
|  | Postural orthostatic tachycardia syndrome | 4 | 0.02 | 5.14(1.92,13.75） | 1.49(0.19,2.78） |
| Respiratory, thoracic and mediastinal disorders | Throat tightness | 32 | 0.19 | 2.02(1.43,2.86） | 0.97(0.47,1.47） |
|  | Pharyngeal swelling | 22 | 0.13 | 2.2(1.45,3.35） | 1.06(0.46,1.66） |
| Ear and labyrinth disorders | Ear discomfort | 14 | 0.08 | 2.2(1.3,3.71） | 1.02(0.27,1.77） |
| Injury, poisoning and procedural complications | Scratch | 20 | 0.12 | 3.17(2.04,4.91） | 1.52(0.88,2.15） |
|  | Concussion | 12 | 0.07 | 2.35(1.33,4.15） | 1.09(0.29,1.89） |
|  | Maternal exposure during breast feeding | 10 | 0.06 | 2.88(1.55,5.36） | 1.29(0.42,2.17） |
| Vascular disorders | Raynaud's phenomenon | 35 | 0.21 | 12.31(8.81,17.21） | 3.21(2.72,3.69） |
| Infections and infestations | Injection site cellulitis | 8 | 0.05 | 8.56(4.26,17.21） | 2.21(1.24,3.18） |
|  | Injection site infection | 7 | 0.04 | 3.97(1.89,8.35） | 1.53(0.5,2.55） |
|  | Injection site pustule | 4 | 0.02 | 25.06(9.21,68.23） | 2.1(0.77,3.42） |
| Total |  | 16,736 | 100 |  |  |

Note：SOC: System Organ Class; PT: preferred term; ROR: reporting odd ratio; IC: information components
