# Supplementary material for: Adverse event reporting of four anti-Calcitonin gene-related peptide monoclonal antibodies for migraine prevention: a real-world study based on the FDA adverse event reporting system
Source: Front Pharmacol. 2024 Jan 9;14:1257282. doi: 10.3389/fphar.2023.1257282 (PMC10803415; doi:10.3389/fphar.2023.1257282)
Supplement: Supplementary file 3 [file Table5.docx]

**Supplementary Table S5.** **Injection-related PT for the four anti-CGRP mAbs**

| PT | erenumab N=16099 | | galcanezumab N=16736 | | fremanezumab N=5886 | | eptinezumab N=1086 | |
| --- | --- | --- | --- | --- | --- | --- | --- | --- |
|  | n | % | n | % | n | % | n | % |
| Injection site pain | 2,267 | 14.08% | 4,079 | 24.37% | 771 | 13.10% |  |  |
| Injection site erythema | 437 | 2.71% | 896 | 5.35% | 413 | 7.02% |  |  |
| Injection site pruritus | 215 | 1.34% | 676 | 4.04% | 322 | 5.47% |  |  |
| Injection site haemorrhage | 926 | 5.75% | 831 | 4.97% | 91 | 1.55% |  |  |
| Injection site swelling | 418 | 2.60% | 663 | 3.96% | 262 | 4.45% |  |  |
| Injection site reaction | 257 | 1.60% | 595 | 3.56% | 193 | 3.28% |  |  |
| Injection site bruising | 535 | 3.32% | 434 | 2.59% | 94 | 1.60% |  |  |
| Injection site urticaria | 158 | 0.98% | 378 | 2.26% | 109 | 1.85% |  |  |
| Injection site mass | 120 | 0.75% | 346 | 2.07% | 131 | 2.23% |  |  |
| Injection site rash | 134 | 0.83% | 270 | 1.61% | 170 | 2.89% |  |  |
| Injection site warmth | 67 | 0.42% | 177 | 1.06% | 76 | 1.29% |  |  |
| Injection site discomfort | 70 | 0.43% | 112 | 0.67% | 18 | 0.31% |  |  |
| Injection site injury | 29 | 0.18% | 104 | 0.62% | 9 | 0.15% |  |  |
| Injection site induration | 22 | 0.14% | 83 | 0.50% | 47 | 0.80% |  |  |
| Injection site irritation | 39 | 0.24% | 69 | 0.41% | 28 | 0.48% |  |  |
| Injection site discolouration | 57 | 0.35% | 50 | 0.30% | 38 | 0.65% |  |  |
| Injection site hypersensitivity | 14 | 0.09% | 42 | 0.25% | 18 | 0.31% |  |  |
| Injection site inflammation | 12 | 0.07% | 34 | 0.20% | 14 | 0.24% |  |  |
| Injection site vesicles | 28 | 0.17% | 32 | 0.19% | 17 | 0.29% |  |  |
| Injection site extravasation | 109 | 0.68% | 23 | 0.14% | 167 | 2.84% |  |  |
| Injection site scar | 9 | 0.06% | 20 | 0.12% | 8 | 0.14% |  |  |
| Injection site haematoma |  |  | 16 | 0.10% |  |  |  |  |
| Injection site nodule |  |  | 15 | 0.09% |  |  |  |  |
| Injection site paraesthesia | 10 | 0.06% | 11 | 0.07% | 5 | 0.08% |  |  |
| Injection site papule | 13 | 0.08% | 11 | 0.07% |  |  |  |  |
| Injection site macule | 6 | 0.04% | 8 | 0.05% |  |  |  |  |
| Injection site cellulitis |  |  | 8 | 0.05% | 8 | 0.14% |  |  |
| Injection site infection |  |  | 7 | 0.04% | 4 | 0.07% |  |  |
| Injection site hypoaesthesia | 18 | 0.11% | 4 | 0.02% | 5 | 0.08% |  |  |
| Injection site laceration |  |  | 4 | 0.02% |  |  |  |  |
| Injection site oedema |  |  | 4 | 0.02% | 8 | 0.14% |  |  |
| Injection site pustule |  |  | 4 | 0.02% |  |  |  |  |
| Injection site coldness | 5 | 0.03% | 3 | 0.02% |  |  |  |  |
| Injection site streaking |  |  | 3 | 0.02% |  |  |  |  |
| Injection related reaction | 17 | 0.11% |  |  |  |  |  |  |
| Injection site indentation | 107 | 0.66% |  |  | 8 | 0.14% |  |  |
| Injection site discharge |  |  |  |  | 52 | 0.88% |  |  |
| Infusion site pain |  |  |  |  |  |  | 23 | 2.12% |
| Infusion related reaction |  |  |  |  |  |  | 19 | 1.75% |
| Infusion site bruising |  |  |  |  |  |  | 11 | 1.01% |
| Infusion site extravasation | |  |  |  |  |  | 9 | 0.83% |
| Infusion site rash |  |  |  |  |  |  | 7 | 0.64% |
| Infusion site swelling |  |  |  |  |  |  | 6 | 0.55% |
| Infusion site pruritus |  |  |  |  |  |  | 6 | 0.55% |
| Infusion site discomfort |  |  |  |  |  |  | 4 | 0.37% |
| Total | 6,099 | 37.88% | 10,012 | 59.82% | 3,086 | 52.43% | 85 | 7.83% |

Note: PT: preferred term
