# Supplementary material for: Adverse event reporting of four anti-Calcitonin gene-related peptide monoclonal antibodies for migraine prevention: a real-world study based on the FDA adverse event reporting system
Source: Front Pharmacol. 2024 Jan 9;14:1257282. doi: 10.3389/fphar.2023.1257282 (PMC10803415; doi:10.3389/fphar.2023.1257282)
Supplement: Supplementary file 4 [file Table3.docx]

**Supplementary Table S3.** PT signal detection under each SOC for Fremanezumab

| SOC | PT | N | % | ROR | IC |
| --- | --- | --- | --- | --- | --- |
| General disorders and administration site conditions | Injection site pain | 771 | 13.10 | 12.53(11.65,13.47) | 3.55(3.45,3.66) |
|  | Injection site erythema | 413 | 7.02 | 20.84(18.89,23.00) | 4.26(4.12,4.41) |
|  | Injection site pruritus | 322 | 5.47 | 26.73(23.92,29.88) | 4.58(4.42,4.74) |
|  | Injection site swelling | 262 | 4.45 | 17.89(15.83,20.23) | 4.04(3.85,4.22) |
|  | Injection site reaction | 193 | 3.28 | 13.74(11.91,15.84) | 3.66(3.45,3.87) |
|  | Injection site rash | 170 | 2.89 | 28.59(24.54,33.30) | 4.59(4.36,4.81) |
|  | Injection site extravasation | 167 | 2.84 | 47.02(40.27,54.89) | 5.16(4.93,5.39) |
|  | Injection site mass | 131 | 2.23 | 11.94(10.04,14.18) | 3.44(3.19,3.70) |
|  | Feeling abnormal | 128 | 2.17 | 2.07(1.74,2.46) | 1.03(0.78,1.29) |
|  | Injection site urticaria | 109 | 1.85 | 21.68(17.93,26.21) | 4.16(3.89,4.44) |
|  | Injection site bruising | 94 | 1.60 | 5.49(4.48,6.73) | 2.38(2.08,2.68) |
|  | Injection site haemorrhage | 91 | 1.55 | 4.68(3.81,5.75) | 2.16(1.86,2.46) |
|  | Injection site warmth | 76 | 1.29 | 25.71(20.48,32.27) | 4.26(3.93,4.59) |
|  | Chest pain | 53 | 0.90 | 1.34(1.02,1.75) | 0.42(0.01,0.80) |
|  | Injection site discharge | 52 | 0.88 | 103.25(77.88,136.89) | 5.10(4.69,5.51) |
|  | Injection site induration | 47 | 0.80 | 18.59(13.93,24.8) | 3.75(3.33,4.17) |
|  | Swelling | 47 | 0.80 | 1.78(1.34,2.37) | 0.83(0.39,1.23) |
|  | Chest discomfort | 44 | 0.75 | 1.73(1.29,2.33) | 0.79(0.34,1.20) |
|  | Swelling face | 39 | 0.66 | 2.49(1.82,3.42) | 1.26(0.80,1.72) |
|  | Injection site discolouration | 38 | 0.64 | 14.41(10.46,19.84) | 3.41(2.94,3.87) |
|  | Feeling hot | 35 | 0.59 | 2.39(1.72,3.33) | 1.20(0.72,1.68) |
|  | Influenza like illness | 34 | 0.58 | 1.94(1.39,2.72) | 0.96(0.43,1.41) |
|  | Injection site irritation | 28 | 0.48 | 17.24(11.87,25.04) | 3.45(2.91,3.99) |
|  | Injection site discomfort | 18 | 0.31 | 6.91(4.34,10.97) | 2.39(1.73,3.06) |
|  | Injection site hypersensitivity | 18 | 0.31 | 24.9(15.62,39.69) | 3.45(2.78,4.12) |
|  | Injection site vesicles | 17 | 0.29 | 13.34(8.27,21.51) | 2.98(2.29,3.66) |
|  | Injection site inflammation | 14 | 0.24 | 13.05(7.71,22.09) | 2.85(2.10,3.60) |
|  | Injection site injury | 9 | 0.15 | 6.82(3.54,13.13) | 2.10(1.19,3.02) |
|  | Injection site oedema | 8 | 0.14 | 20.87(10.38,41.95) | 2.70(1.73,3.67) |
|  | Injection site scar | 8 | 0.14 | 10.96(5.47,21.99) | 2.37(1.41,3.34) |
|  | Injection site indentation | 8 | 0.14 | 10.76(5.37,21.58) | 2.36(1.40,3.33) |
|  | Immediate post-injection reaction | 7 | 0.12 | 24.79(11.74,52.35) | 2.63(1.60,3.67) |
|  | Injection site paraesthesia | 5 | 0.08 | 14.34(5.94,34.62) | 2.15(0.96,3.33) |
|  | Injection site hypoaesthesia | 5 | 0.08 | 12.60(5.22,30.39) | 2.10(0.91,3.28) |
|  | Induration | 4 | 0.07 | 10.98(4.11,29.38) | 1.87(0.57,3.17) |
| Skin and subcutaneous tissue disorders | Pruritus | 216 | 3.67 | 2.19(1.91,2.50) | 1.11(0.91,1.31) |
|  | Rash | 190 | 3.23 | 2.19(1.91,2.50) | 1.12(0.91,1.31) |
|  | Alopecia | 182 | 3.09 | 2.73(2.36,3.16) | 1.42(1.21,1.64) |
|  | Urticaria | 132 | 2.24 | 3.19(2.69,3.79) | 1.64(1.39,1.89) |
|  | Erythema | 85 | 1.44 | 1.27(1.02,1.57） | 0.34(0.02,0.65) |
|  | Rash pruritic | 31 | 0.53 | 2.42(1.70,3.44) | 1.21(0.70,1.72) |
|  | Rash erythematous | 19 | 0.32 | 1.77(1.13,2.78) | 0.82(0.12,1.41) |
|  | Cold sweat | 9 | 0.15 | 2.29(1.19,4.40) | 1.02(0.10,1.93) |
|  | Skin reaction | 9 | 0.15 | 2.34(1.21,4.49) | 1.04(0.13,1.95) |
|  | Trichorrhexis | 6 | 0.10 | 8.74(3.92,19.51) | 2.05(0.95,3.14) |
|  | Skin induration | 3 | 0.05 | 6.66(2.14,20.71) | 1.46(0.01,2.91) |
|  | Needle track marks | 3 | 0.05 | 35.74(11.36,112.41) | 1.88(0.41,3.35) |
| Psychiatric disorders | Anxiety | 122 | 2.07 | 1.64(1.38,1.97) | 0.71(0.44,0.97) |
|  | Insomnia | 93 | 1.58 | 1.52(1.24,1.87) | 0.60(0.30,0.89) |
|  | Panic attack | 34 | 0.58 | 4.23(3.02,5.93) | 1.95(1.46,2.44) |
|  | Nervousness | 21 | 0.36 | 1.84(1.20,2.83) | 0.88(0.21,1.44) |
|  | Nightmare | 14 | 0.24 | 1.96(1.16,3.32) | 0.97(0.14,1.63) |
|  | Fear of injection | 12 | 0.20 | 5.79(3.28,10.21) | 2.08(1.27,2.88) |
|  | Fear | 11 | 0.19 | 2.42(1.34,4.37) | 1.11(0.28,1.94) |
|  | Abnormal dreams | 10 | 0.17 | 2.37(1.27,4.41) | 1.07(0.20,1.94) |
|  | Near death experience | 8 | 0.14 | 5.88(2.94,11.78) | 1.93(0.96,2.89) |
|  | Panic reaction | 8 | 0.14 | 4.89(2.44,9.79) | 1.77(0.80,2.73) |
|  | Inappropriate affect | 3 | 0.05 | 9.07(2.91,28.22) | 1.58(0.14,3.03) |
| Gastrointestinal disorders | Constipation | 159 | 2.70 | 2.86(2.45,3.35) | 1.49(1.26,1.72) |
|  | Abdominal distension | 37 | 0.63 | 1.50(1.08,2.07) | 0.58(0.09,1.03) |
|  | Swollen tongue | 26 | 0.44 | 3.84(2.62,5.65) | 1.79(1.24,2.35) |
|  | Irritable bowel syndrome | 11 | 0.19 | 2.16(1.20,3.90) | 0.98(0.14,1.81) |
|  | Paraesthesia oral | 11 | 0.19 | 3.19(1.77,5.77) | 1.43(0.60,2.26) |
|  | Mesenteric panniculitis | 3 | 0.05 | 43.03(13.64,135.75) | 1.90(0.43,3.37) |
| Musculoskeletal and connective tissue disorders | Arthralgia | 146 | 2.48 | 1.33(1.13,1.57) | 0.41(0.17,0.65) |
|  | Myalgia | 66 | 1.12 | 1.66(1.31,2.12) | 0.73(0.36,1.07) |
|  | Muscle tightness | 11 | 0.19 | 2.92(1.62,5.28) | 1.33(0.50,2.16) |
| Nervous system disorders | Paraesthesia | 58 | 0.99 | 1.54(1.19,1.99) | 0.62(0.23,0.98) |
|  | Hypoaesthesia | 56 | 0.95 | 1.58(1.22,2.06) | 0.66(0.26,1.03) |
|  | Burning sensation | 34 | 0.58 | 2.04(1.46,2.86) | 0.98(0.50,1.47) |
|  | Speech disorder | 29 | 0.49 | 2.32(1.61,3.35) | 1.15(0.62,1.68) |
|  | Taste disorder | 14 | 0.24 | 1.7791.05,2.99） | 0.82(0.00,1.50） |
|  | Formication | 5 | 0.08 | 3.45(1.43,8.29) | 1.29(0.11,2.47) |
|  | Monoplegia | 4 | 0.07 | 3.96(1.48,10.57) | 1.31(0.02,2.61) |
|  | Cerebellar infarction | 3 | 0.05 | 9.19(2.95,28.59) | 1.59(0.14,3.04) |
| Investigations | Weight increased | 139 | 2.36 | 2.51(2.12,2.96) | 1.30(1.06,1.55) |
|  | Hormone level abnormal | 5 | 0.08 | 3.89(1.62,9.37) | 1.39(0.21,2.57) |
| Immune system disorders | Hypersensitivity | 112 | 1.90 | 2.14(1.78,2.58) | 1.08(0.80,1.35) |
| Cardiac disorders | Palpitations | 85 | 1.44 | 3.09(2.50,3.83) | 1.59(1.27,1.90) |
| Injury, poisoning and procedural complications | Contusion | 37 | 0.63 | 1.56(1.13,2.15) | 0.64(0.15,1.08) |
|  | Injection related reaction | 9 | 0.15 | 16.83(8.72,32.48) | 2.70(1.78,3.62) |
|  | Scratch | 8 | 0.14 | 3.15(1.57,6.30) | 1.34(0.38,2.31) |
|  | Thermal burn | 6 | 0.10 | 3.74(1.68,8.33) | 1.42(0.33,2.52) |
|  | Needle fatigue | 3 | 0.05 | 17.87(5.72,55.81) | 1.77(0.32,3.23) |
| Respiratory, thoracic and mediastinal disorders | Throat tightness | 29 | 0.49 | 4.57(3.17,6.59) | 2.03(1.50,2.55) |
|  | Pharyngeal swelling | 12 | 0.20 | 2.99(1.70,5.26) | 1.37(0.57,2.17) |
|  | Oropharyngeal discomfort | 7 | 0.12 | 2.41(1.15,5.05) | 1.03(0.01,2.05) |
|  | Pharyngeal paraesthesia | 5 | 0.08 | 8.45(3.51,20.35) | 1.91(0.73,3.09) |
| Reproductive system and breast disorders | Menstrual disorder | 14 | 0.24 | 7.35(4.35,12.43) | 2.36(1.62,3.11) |
|  | Menstruation irregular | 9 | 0.15 | 2.98(1.55,5.73) | 1.31(0.40,2.23) |
|  | Polymenorrhoea | 6 | 0.10 | 9.42(4.22,21.04) | 2.09(1.00,3.19) |
|  | Oligomenorrhoea | 3 | 0.05 | 8.48(2.73,26.40) | 1.56(0.11,3.01) |
| Vascular disorders  Eye disorders | Raynaud's phenomenon | 14 | 0.24 | 12.12(7.16,20.51) | 2.79(2.04,3.54) |
|  | Asthenopia | 6 | 0.10 | 5.23(2.34,11.65) | 1.70(0.61,2.79) |
|  | Blepharospasm | 6 | 0.10 | 5.00(2.24,11.14) | 1.67(0.57,2.76) |
| Infections and infestations | Injection site cellulitis | 8 | 0.14 | 21.34(10.62,42.91) | 2.70(1.73,3.67) |
|  | Injection site infection | 4 | 0.07 | 5.64(2.11,15.06) | 1.55(0.25,2.84) |
| Total |  | 5886 | 100 |  |  |

Note：SOC: System Organ Class; PT: preferred term; ROR: reporting odd ratio; IC: information components
