# Supplementary material for: Adverse event reporting of four anti-Calcitonin gene-related peptide monoclonal antibodies for migraine prevention: a real-world study based on the FDA adverse event reporting system
Source: Front Pharmacol. 2024 Jan 9;14:1257282. doi: 10.3389/fphar.2023.1257282 (PMC10803415; doi:10.3389/fphar.2023.1257282)
Supplement: Supplementary file 5 [file Table4.docx]

**Supplementary Table S4.** PT signal detection under each SOC for Eptinezumab

| SOC | PT | n | % | ROR | CI |
| --- | --- | --- | --- | --- | --- |
| General disorders and administration site conditions | Fatigue | 147 | 13.54 | 2.25(1.91,2.65) | 1.14(0.89,1.38) |
|  | Feeling abnormal | 61 | 5.62 | 3.16(2.45,4.06) | 1.60(1.23,1.97) |
|  | Chest discomfort | 24 | 2.21 | 3.02(2.02,4.51) | 1.48(0.9,2.06) |
|  | Infusion site pain | 23 | 2.12 | 20.31(13.47,30.62) | 3.49(2.89,4.08) |
|  | Infusion site bruising | 11 | 1.01 | 58.27(32.13,105.7) | 3.33(2.49,4.17) |
|  | Infusion site extravasation | 9 | 0.83 | 13.09(6.8,25.2) | 2.56(1.65,3.48) |
|  | Infusion site rash | 7 | 0.64 | 62.35(29.56,131.52) | 2.84(1.82,3.87) |
|  | Feeling cold | 7 | 0.64 | 3.30(1.57,6.93) | 1.36(0.34,2.38) |
|  | Infusion site swelling | 6 | 0.55 | 10.04(4.5,22.38) | 2.13(1.04,3.22) |
|  | Infusion site pruritus | 6 | 0.55 | 21.75(9.75,48.53) | 2.45(1.36,3.55) |
|  | Infusion site discomfort | 4 | 0.37 | 41.54(15.51,111.23) | 2.19(0.89,3.49) |
| Respiratory, thoracic and mediastinal disorders | Throat irritation | 98 | 9.02 | 29.51(24.15,36.06) | 4.49(4.20,4.78) |
|  | Nasal congestion | 81 | 7.46 | 17.05(13.69,21.25) | 3.81(3.49,4.13) |
|  | Oropharyngeal pain | 45 | 4.14 | 5.79(4.32,7.77) | 2.38(1.95,2.81) |
|  | Rhinorrhoea | 40 | 3.68 | 6.90(5.05,9.41) | 2.58(2.13,3.03) |
|  | Sneezing | 20 | 1.84 | 11.3(7.28,17.54) | 2.92(2.29,3.55) |
|  | Throat tightness | 13 | 1.20 | 6.53(3.79,11.26) | 2.22(1.45,3.00) |
|  | Sinus congestion | 8 | 0.74 | 8.31(4.15,16.64) | 2.19(1.23,3.16) |
|  | Pharyngeal swelling | 8 | 0.74 | 6.35(3.17,12.72) | 1.99(1.03,2.96) |
|  | Upper-airway cough syndrome | 4 | 0.37 | 5.49(2.06,14.63) | 1.53(0.24,2.82) |
|  | Paranasal sinus discomfort | 3 | 0.28 | 8.39(2.7,26.05) | 1.56(0.11,3) |
|  | Nasal pruritus | 3 | 0.28 | 25.39(8.16,79) | 1.84(0.39,3.29) |
| Infections and infestations | COVID-19 | 61 | 5.62 | 2.39(1.86,3.07) | 1.21(0.84,1.58) |
|  | Nasopharyngitis | 31 | 2.85 | 1.98(1.39,2.81) | 0.98(0.94,0.42) |
| Skin and subcutaneous tissue disorders | Pruritus | 89 | 8.20 | 2.88(2.34,3.55) | 1.48(1.17,1.79) |
| Immune system disorders | Hypersensitivity | 56 | 5.16 | 3.43(2.63,4.46) | 1.70(1.32,2.09) |
|  | Anaphylactic reaction | 21 | 1.93 | 4.95(3.22,7.60) | 2.06(1.45,2.68) |
|  | Seasonal allergy | 5 | 0.46 | 3.78(1.57,9.1) | 1.37(0.19,2.55) |
| Gastrointestinal disorders | Constipation | 31 | 2.85 | 1.77(1.24,2.52) | 0.82(0.79,0.28) |
|  | Dry mouth | 13 | 1.20 | 2.29(1.33,3.95) | 1.07(0.30,1.84) |
|  | Paraesthesia oral | 9 | 0.83 | 8.34(4.34,16.05) | 2.26(1.35,3.18) |
|  | Swollen tongue | 6 | 0.55 | 2.82(1.27,6.29) | 1.16(0.07,2.25) |
| Vascular disorders | Flushing | 22 | 2.03 | 3.53(2.32,5.37) | 1.66(1.06,2.27) |
|  | Hot flush | 12 | 1.10 | 2.10(1.19,3.70) | 0.95(0.15,1.75) |
| Injury, poisoning and procedural complications | Infusion related reaction | 19 | 1.75 | 3.34(2.13,5.23) | 1.57(0.93,2.22) |
|  | Concussion | 6 | 0.55 | 9.34(4.19,20.82) | 2.09(1.00,3.18) |
|  | Ligament sprain | 4 | 0.37 | 4.59(1.72,12.23) | 1.42(0.12,2.71) |
| Nervous system disorders | Memory impairment | 25 | 2.30 | 2.14(1.45,3.18) | 1.03(0.47,1.60) |
|  | Electric shock sensation | 4 | 0.37 | 16.9(6.33,45.12) | 2.01(0.72,3.31) |
| Eye disorders | Eye pruritus | 8 | 0.74 | 3.03(1.51,6.06) | 1.30(0.34,2.27) |
|  | Blepharospasm | 4 | 0.37 | 10.62(3.98,28.33) | 1.86(0.57,3.15) |
| Investigations | Heart rate increased | 17 | 1.57 | 2.26(1.40,3.64) | 1.08(0.39,1.76) |
| Musculoskeletal and connective tissue disorders | Fibromyalgia | 9 | 0.83 | 4.90(2.55,9.43) | 1.82(0.90,2.73) |
|  |  |  |  |  |  |
| Metabolism and nutrition disorders | Increased appetite | 6 | 0.55 | 5.02(2.25,11.19) | 1.67(0.58,2.76) |
| Total |  | 1,086 | 100 |  |  |

Note：SOC: System Organ Class; PT: preferred term; ROR: reporting odd ratio; IC: information components
